# Gingival Displacement in the Vertical and Horizontal Dimension under the Condition of Mild Gingivitis—A Randomized Clinical Study

**DOI:** 10.3390/jcm11020437

**Published:** 2022-01-15

**Authors:** Katharina Kuhn, David Zügel, Victor-Sebastian A. Korbay, Thomas Papas, Sigmar Schnutenhaus, Ralph G. Luthardt, Jens Dreyhaupt, Heike Rudolph

**Affiliations:** 1 Department of Prosthetic Dentistry, Center of Dentistry, Ulm University, Albert-Einstein-Allee 11, 89081 Ulm, Germany; drzuegel@zahnarztpraxis-lenggries.de (D.Z.); v.korbay@gmx.de (V.-S.A.K.); thomaspapas99@yahoo.de (T.P.); sigmar.schnutenhaus@uniklinik-ulm.de (S.S.); computerzaehne@computerzaehne.de (R.G.L.); heike.rudolph@computerzaehne.de (H.R.); 2 Institute of Epidemiology and Medical Biometry, Ulm University, Schwabstraße 13, 89075 Ulm, Germany; jens.dreyhaupt@uni-ulm.de

**Keywords:** gingival displacement, double cord technique, aluminum chloride paste, experimentally induced gingivitis, digitization, computer-assisted analysis

## Abstract

This randomized clinical study aimed at quantifying the gingival displacement performance in the vertical and horizontal directions of the 3M™ Astringent Retraction Paste (3M Oral Care, Seefeld, Germany) in comparison with the double-cord technique with aluminum chloride as an astringent. Afterward, any soft-tissue changes were assessed for 12 months. After inducing mild gingivitis, 18 probands received the intervention ‘cord’ and 22 probands received the intervention ‘paste’ at the palatal half of upper premolars prior to conventional impression making. The resulting plaster casts were digitized and analyzed for the vertical and horizontal gingival displacement, applying a newly developed computer-assisted methodology. The entire palatal half of the tooth was evaluated instead of only single sites. Under the condition of mild gingivitis, the gingival displacement performance was comparable for both techniques in the horizontal direction (width) and only somewhat better for the cord technique in the vertical direction (depth). The magnitude of displacement was in a similar range in both directions, with somewhat higher values in the vertical direction. The marginal gingiva height changes were of such low extent during the follow-up period of 12 months with only minimally higher values for the paste that they cannot be considered as clinically relevant recessions.

## 1. Introduction

Adequate gingival displacement both in the vertical and horizontal direction is a mandatory requirement before impression making of subgingivally prepared teeth for fixed dental prostheses [1]. Next to the cord technique, there is a variety of different cordless systems for gingival displacement, such as pastes, foams and gels [2]. The comparison of the currently well-known cordless techniques to the conventional cord technique generally showed superior gingival displacement performance for the cord technique under healthy gingival conditions [3,4,5,6,7,8]. However, it is necessary to differentiate between the different cordless systems. While an addition-curing silicone foam (Magic Foam Cord, Coltène Whaledent, Altstätten, Switzerland) showed significantly less horizontal gingival displacement compared to the cord technique, the somewhat worse performance of a kaolin paste system (Expasyl, Pierre Rolland, Merignac, France) compared to the cord technique did not reach statistical significance in a recent meta-analysis [7]. While these two cordless systems have been investigated in several studies, the 3M™ Astringent Retraction Paste (3M Oral Care, Seefeld, Germany), a capsule-based system with less than 5% kaolin and 15% aluminum chloride hexahydrate [9], has barely been studied. The little clinical evidence indicates a superiority of this system: it showed significantly more horizontal displacement than Expasyl under healthy gingival conditions [10]. A follow-up report [11] and survey [9] showed a high usability in the clinical routine probably due to the thin application tips and the suitable viscosity [9]. However, the number of studies is not yet sufficient to draw a concluding statement regarding its gingival displacement performance in the vertical and horizontal directions.

Another crucial requirement for gingival displacement techniques is the avoidance of permanent gingival recessions. Paste systems were more successful in achieving this goal than cord systems [5,12,13,14,15]. To the authors’ best knowledge, apart from a follow-up report with promising results [11], no studies reporting on gingival height changes have been performed for the 3M™ Astringent Retraction Paste. In an in vitro study, this paste system resulted in significantly less pressure in an artificial sulcus than the well-studied system Expasyl [16]. Clinical data describing marginal height changes are still missing. However, no relevance for the multifactorial-influenced consequences of gingival displacement procedures can be derived from these in vitro results.

In most studies, the follow-up period for the investigation of the gingival height changes are only three months or less after the intervention impression [12,13,14,15]. As a loss of the marginal gingiva height was still recorded from the one-month to three-month follow-up appointment [13], longer clinical follow-up procedures seem advisable to exclude the formation of permanent recessions.

The gingival conditions have to be considered when investigating gingival displacement techniques. In the clinical reality, the inclusion criteria of a healthy periodontal and/or gingival status as given in clinical studies [3,4,5,7,8,10,12,17,18,19,20,21,22,23,24,25] are often not fully met. 

One of the many reasons for this is that the impression is often made of more abutment teeth simultaneously in everyday clinical situations. The only one or two abutment teeth in most clinical studies are more easily controllable in terms of hygiene, especially under study conditions. Thus, mild gingivitis—sometimes only at one site of one abutment tooth—is frequently present in the clinical reality. Another scenario in which gingivitis may occur is the following: if the impression is made in the following appointment after the preparation session, a provisional acrylic crown is worn in the meantime. These restorations are known to favor plaque accumulation [26], which may lead to gingivitis by the time of impression making.

In a comparative study of cord versus kaolin paste system (Expasyl) performance, in which mild gingivitis was induced on purpose, the gingival condition worsened the cord’s performance but did not influenced the paste’s condition [6]. This clinically highly relevant influencing factor was not investigated for further paste systems, especially not for the 3M™ Astringent Retraction Paste. Its ingredient aluminum chloride is known for effective hemostasis and drying of the sulcus [27]. This may qualify the paste system as a particularly suitable material for abutment teeth with mild gingivitis. Data of a survey among dentists support this thesis, as 75% claimed the 3M™ Astringent Retraction Paste to have good hemostatic properties [9]. However, clinical data on the performance of this paste system under the defined condition of mild gingivitis are missing.

Due to the potential of the 3M™ Astringent Retraction Paste, which can be deduced from the limited available literature, the scientific task was to systematically investigate its performance. The aim of the study was to quantify its vertical (sulcus depth) and horizontal (sulcus width) gingival displacement performance in comparison with the gold standard (double cord technique) under the condition of mild gingivitis and to record a possible marginal gingiva height change or recession over an extended period of time (up to 12 months).

## 2. Materials and Methods

The randomized clinical trial was approved by the Ethics Committee of Ulm University (number: 310/14) on 3 December 2014, and has been prospectively registered in the World Health Organization’s International Clinical Trials Registry with the main ID number DRKS00007809. The recruitment took place from February 2015 until November 2016 to include the intended number of subjects (*n* = 40). The last follow-up visit (last patient out) was performed in December 2017.

### 2.1. Trial Design

**Figure 1:**
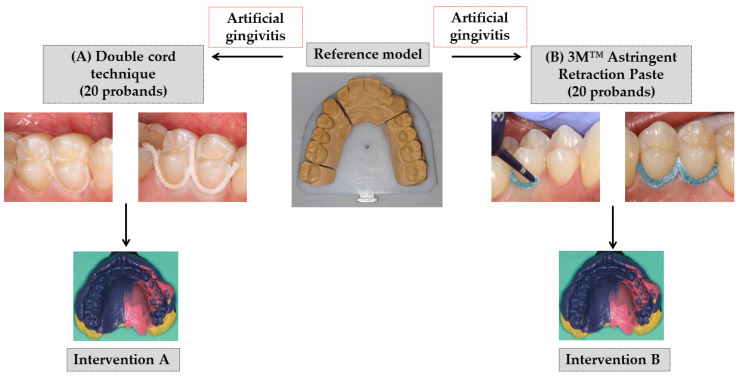
Design of the randomized clinical trial (RCT) with the two interventions: (**A**) double-cord technique or (**B**) 3M™ Astringent Retraction Paste for gingival displacement prior to conventional impression making.

**Figure 2:**
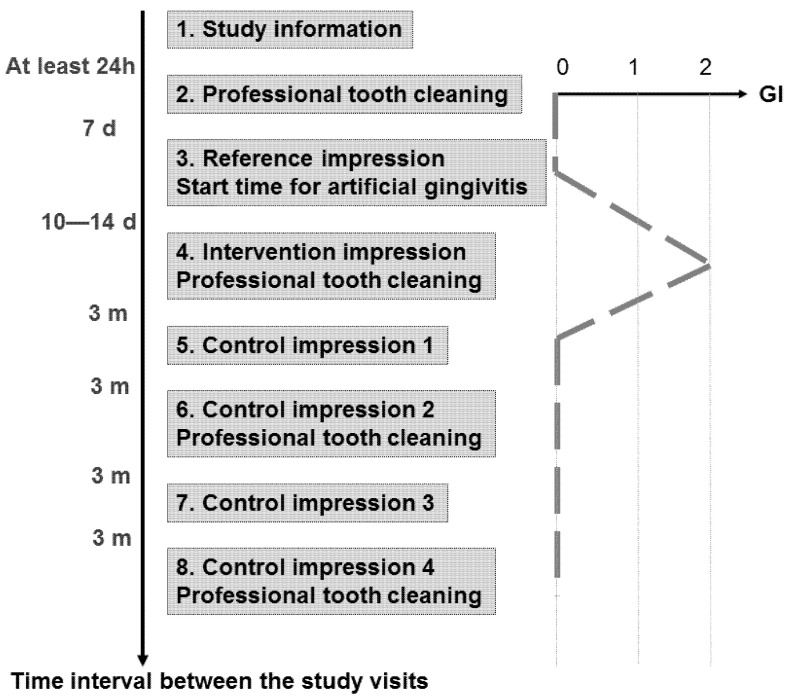
Study visits with intended course of the gingival index (GI). h: hours; d: days; m: months. Adapted with permission from [6]. 2021 MDPI.

Few basic elements of the concept of a previous study [6], performed at the same study center, were used for this study design (Figure 1). For the intervention B, the paste (product in previous study: Expasyl, Pierre Rolland, Merignac, France) was replaced by the paste product 3M™ Astringent Retraction Paste (3M Oral Care, Seefeld, Germany). The comparison was again made for the double cord technique (Retracto, Roeko, Langenau, Germany) in combination with the astringent aluminum chloride (intervention A). Focusing on the outcomes under the condition of mild gingivitis, the cross-over comparison (first intervention for healthy gingival conditions) of the preceding study was omitted. In the study presented here, the artificial gingivitis was induced immediately after the baseline impression. Furthermore, the follow-up period was extended from 6 to 12 months (Figure 2).

### 2.2. Participants

The inclusion and exclusion criteria were in close accordance with those already described for the previous study [6].

Inclusion criteria:

- Subjects aged 18 to 80 years;
- Healthy (no chronic or acute infections or diseases);
- Periodontal Screening Index (PSI; definition by the German Society for Periodontology) at or below 2;
- Presence of both premolars in the upper jaw with their adjacent teeth;
- Naturally healthy upper premolars and adjacent teeth or restored without defects with indirect or direct restoration margins located at least 1 mm supragingival;
- Legally competent.

Exclusion criteria:

- Pregnancy;
- Alcohol or drug abuse;
- Smoker;
- Allergies to the materials used (astringent, anesthetic, impression material).

The clinical investigators at the study center (Department of Prosthetic Dentistry, Center of Dentistry at Ulm University in Germany) obtained informed consent for all probands and their privacy rights were respected. The study was carried out in accordance with the Code of Ethics of the World Medical Association (Declaration of Helsinki).

### 2.3. Interventions

Eight study visits were performed by two investigators within approximately 13 months for each proband (Figure 2). We obtained informed consent at least 24 h after study information (Visit 1). At Visit 2, the pocket depths were measured (6-point measurement). Professional tooth cleaning was performed with subsequent fluoridation and individual hygiene instructions were given to achieve a gingival index (GI, [28]) of zero at Visit 3. At that appointment, a reference impression was made without performing any gingival displacement after carefully removing any plaque, if present. The trays were individually customized for each proband with a “stop” at the palate made by condensation-curing silicone (Optosil Plus, Heraeus Kulzer, Hanau, Germany). In this way, we ensured enough pressure while excluding too much pressure during the following precision impression. For this precision impression applying the one-stage two-phase impression technique, a polyether material (Impregum Penta H DuoSoft Quick (Heavy Body) and Impregum Garant L DuoSoft Quick (Light Body), 3M Oral Care, Seefeld, Germany) was used. At the end of this appointment, the proband was instructed to abandon any oral hygiene measures until Visit 4, which took place 10 to 14 days later.

Consequently, mild gingivitis with an intended GI of 2 should be established at Visit 4, when the intervention impression was made (Figure 1). According to the allocated randomization group, we performed the gingival displacement technique A or B at the palatal premolars’ sites of the first or the second quadrant. 

For intervention A, the same procedure was performed as described in detail in the publication of the prior study [6]. Briefly summarized, a primary cord of size 1 (Retracto, impregnated, Roeko, Langenau, Germany) followed by a secondary cord of size 2 (Retracto, not impregnated, Roeko, Langenau, Germany) were applied on the premolars’ palatal side from papilla to papilla. After ten minutes, the primary and secondary cords were removed after thoroughly wetting them to reduce the risk of disrupting the epithelium, and the light body polyether material followed by the heavy body polyether material (see reference impression above) were applied for impression making.

For intervention B, the 3M™ Astringent Retraction Paste (3M Oral Care, Seefeld, Germany) was applied on the palatal side from papilla to papilla. The prefabricated disposable capsules were used with a conventional composite application gun. The paste also remained in the sulcus for ten minutes and was thoroughly rinsed with water spray before impression making. 

Repetition of an intervention impression was not allowed. Thus, incorrect intervention impressions would have led to censoring of the case (drop-out). This did not take place in the study. All impressions were disinfected, blinded, anonymized and further processed. The fabrication of models was performed according to the Giroform process (Amann Girrbach, Pforzheim, Germany) with segmentations between individual tooth segments in the dental arch to minimize the plaster expansion. The resulting plaster saw-cut models were digitized for the gingival displacement and marginal gingiva height analyses (see further below). After intervention impression A or B, a professional tooth cleaning was performed with the aim of a GI of zero at the next appointment.

The first follow-up visit (Visit 5) took place three months later. Supragingival plaque was carefully removed if present prior to the first control impression applying the one-stage two-phase technique without any gingival displacement measures analogous to the reference impression. The impression was processed as described above, and the resulting first control model was also digitized.

The subsequent follow-up visits (Visits 6–8), with a time interval of three months to each other, were performed in the same way as the first one (Visit 5). At Visit 6 and 8, we performed professional tooth cleaning at the end of the appointment.

### 2.4. Outcomes

The primary outcome was the vertical gingival displacement, as defined below in Section 2.4.1. The secondary outcomes were the horizontal gingival displacement (see Section 2.4.2) and marginal gingiva height (see Section 2.4.3). All outcomes are based on three-dimensional (3D) analyses. The prior digitization process and the data processing as well as the 3D analyses are similar in basic principles to the methods described before [6] but have been notably modified and extended for this RCT. For both gingival displacement analyses (depth and width), the digital datasets of the reference and the intervention model had to be aligned to each other to determine differences (in mm). For the marginal gingiva height change analyses (in mm), the datasets of the intervention model and the four control models had to be aligned to the respective reference model.

For all outcomes, the reference, intervention and control models had to be digitized first. After digitization, the premolars’ palatal sulci of the reference and intervention models were exposed for the primary outcome analyses. Therefore, the sulci were carefully undercut by a ball-shaped diamond (diameter 0.8 mm) with the aid of magnifying glasses (magnification 3.5×). A breaking point was provoked at the deepest point of the sulci, leading to exposure. The exposed models were digitized again. We used a non-contact optical digitizing method (DigiSCAN L, AmannGirrbach, Pforzheim, Germany; measurement uncertainty ~16 μm). System-specific filtering was performed to increase data quality by excluding outliers and scatter points (Argus, Fraunhofer Institut IOF, Jena, Germany). All datasets were checked for quality by the supervisor of the study after digitization and faulty digitizations had to be repeated. The unexposed reference and intervention models were digitized twice—(1) with an occlusal and (2) with a palatal orientation between model and camera. With the occlusal orientation, the model was optimally aligned for the horizontal gingival displacement analyses (sulcus width). The palatal orientation was optimal for the vertical gingival displacement analyses (sulcus depth) and the marginal gingiva height analyses (recession). Therefore, the control models were also digitized with this orientation. Subsequently, these datasets were aligned to each other (geomagic studio^®^ + qualify^®^, geomagic Inc., Research Triangle Park, NC, USA) in a common analysis coordinate system (best-fit registration). The datasets of the unexposed intervention model and the four control models all were aligned to the corresponding unexposed reference model dataset. As an indicator for the quality of the registration, the root mean square (RMS) error was determined. It should be less than 32 µm for complete arch models corresponding to the measurement uncertainty of the digitizing system (according to the manufacturer’s specifications ~16 µm). After registration, the 3D analyses for the primary and the two secondary outcomes followed.

#### 2.4.1. Vertical Gingival Displacement

**Figure 3:**
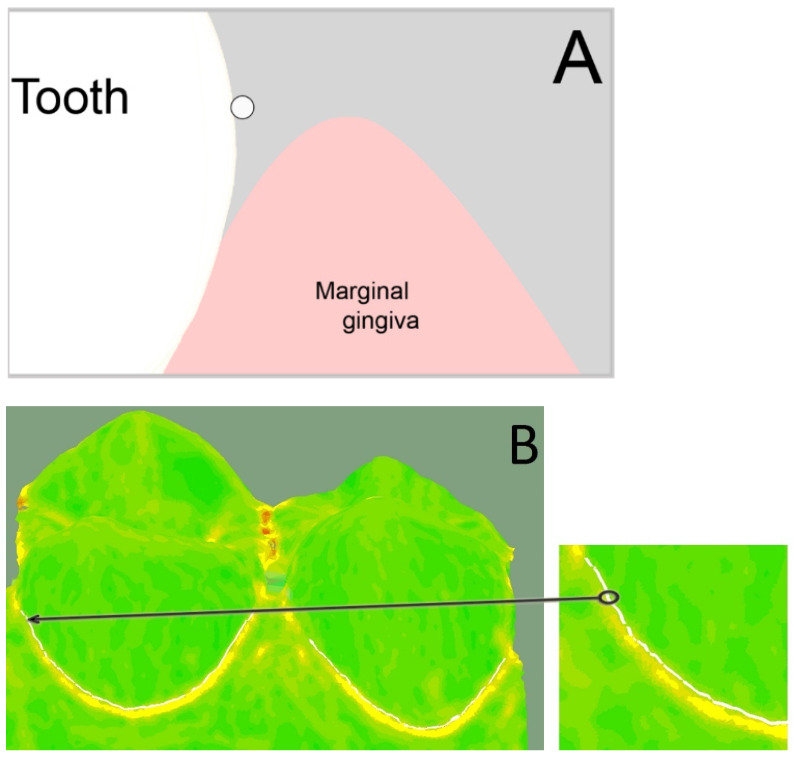
(**A**) Position of base curve (white dot) at the palatal dental cervix. Schematic view in cross section. (**B**) Creation of base curve (white line) at the transition from convex (color: green) to concave (color: yellow) on the palatal dental cervix of the premolars.

For the 3D analyses, three boundary curves had to be created for each premolar in the analysis software (Surfacer^®^ 10.6, SDRC Imageware, Ann Arbor, MI, USA). The (1) base curve was created on the palatal dental cervix and marked the entrance into the sulcus. Therefore, the diagnosis tool “curvature” was used. This tool encodes the transition from convex (color: green) to concave (color: yellow) in color. The base curve was created at this transition using the tool “interactive 3D B-spline” (Figure 3). 

The (2) reference sulcus curve was created at the bottom of the sulcus of the exposed reference model and the (3) intervention sulcus curve was created at the bottom of the sulcus of the exposed intervention model. Therefore, the diagnosis tool “curvature” was used again to mark the transition at the change of curvature. 

**Figure 4:**
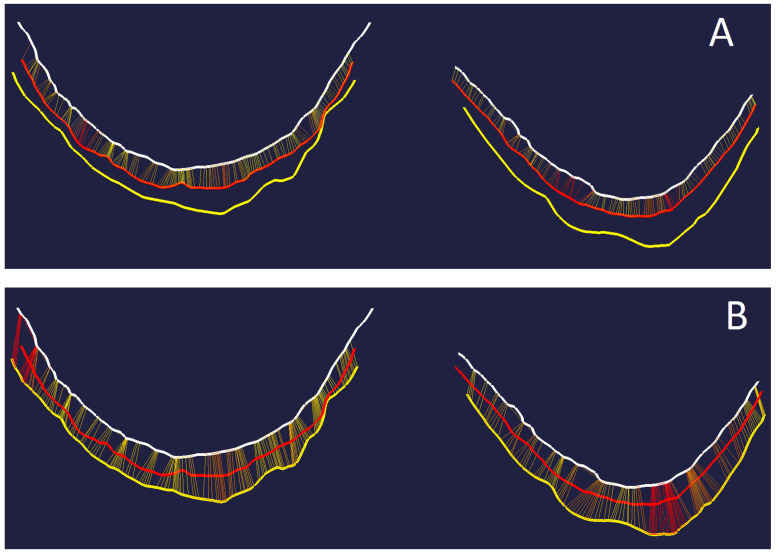
(**A**) Difference between base curve (white) and reference sulcus (RefS) curve (red) on both premolars (datasets of premolars hidden), resulting in the absolute sulcus depth without any gingival displacement. (**B**) Difference between base curve (white) and intervention sulcus (IntS) curve (yellow) on both premolars (datasets of premolars hidden), resulting in the absolute sulcus depth after gingival displacement by cord or paste.

The difference (in mm) between (1) base curve and (2) reference sulcus curve was automatically calculated using the diagnosis tool “Curve-Curve-Difference”. This value represented the absolute sulcus depth captured by the reference impression without any gingival displacement (Figure 4A). The same was calculated for the difference between (1) base curve and (3) intervention sulcus curve, which represented the absolute sulcus depth after gingival displacement by cord or paste captured by the intervention impression (Figure 4B). The vertical gingival displacement was calculated by taking the difference between the two values of the absolute sulcus depths.

We compared the absolute sulcus depth after gingival displacement (Figure 4B) between this study and the prior study [6]. As the absolute sulcus depth was not determined in the prior study, we applied the new method to the still available model datasets of the prior study acquired under the same conditions (mild gingivitis). As intervention A (cords) was identical in both studies, we expected comparable values. Possible differences between both pastes (intervention B: Expasyl [6] versus 3M™ Astringent Retraction Paste) were to be detected.

#### 2.4.2. Horizontal Gingival Displacement

**Figure 5:**
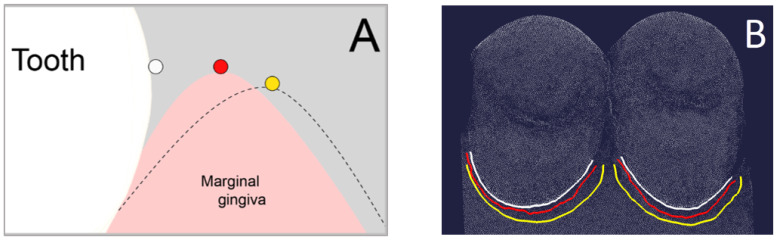
(**A**) Position of base curve (white dot), reference marginal curve (red dot) and intervention marginal curve after gingival displacement (yellow dot). Schematic view in cross section. (**B**) Base curve (white), reference marginal curve (red) and intervention marginal curve (yellow) on both premolars after aligning the datasets of unexposed reference and intervention model.

For the horizontal gingival displacement measurements, three boundary curves (splines) had to be defined at the palatal half of each premolar as well. The (1) base curve was determined as described above for the vertical gingival displacement. The (2) reference marginal curve and the (3) intervention marginal curve were created along the gingival margin (Figure 5) of the unexposed reference model and unexposed intervention model, respectively. Therefore, the diagnosis tool “curvature” was used again to mark the change of curvature.

The difference (in mm) between (1) base curve and (2) reference marginal curve was automatically calculated using the diagnosis tool “Curve-Curve-Difference”. This value represented the absolute sulcus width captured by the reference impression without any gingival displacement. The same was calculated for the difference between (1) base curve and (3) intervention marginal curve, which represented the absolute sulcus width after gingival displacement by cord or paste captured by the intervention impression. The horizontal gingival displacement was calculated by taking the difference between the two values of the absolute sulcus width.

#### 2.4.3. Marginal Gingiva Height

For the marginal gingiva height change analyses, the same procedure as described for the prior study [6] was applied. Hard tissue points were created at the palatal cusp tip of both premolars. Those points were transferred from the reference model dataset to the superimposed intervention and control model datasets. Soft tissue points were created at the deepest point of the marginal gingiva and the distance between hard and soft tissue point was measured (Geomagic Studio^®^ 9 and Qualify^®^ 9, Geomagic Inc., Research Triangle Park, NC, USA) for each model dataset. The marginal gingiva height change was calculated by subtracting those measured distances (reference model minus intervention/control model). Using the reference model, which was taken at Visit 3 seven days after having performed professional tooth cleaning (Figure 2), ensured sound gingival conditions without swelling. In contrast to the prior study, the intervention models were also superimposed. Thus, the displacement of the gingival margin in apical direction could be determined immediately after the gingival displacement procedure. Moreover, four control models with a time interval of three months to each other instead of only two control models were superimposed. Thus, the marginal gingiva height change could be evaluated up to 12 months instead of only six months after intervention in the prior study [6].

### 2.5. Sample Size

A formal sample size calculation based on the prior study [6] was not possible for the following reasons. The study designs differed from each other as well as one of the intervention materials (paste). Furthermore, the primary outcome was measured via a modified method. Considering the experience from similar studies, we set the number of cases at 40 subjects for this explorative pilot study. 

### 2.6. Randomization

The block randomization was performed by a staff member, who was otherwise not involved in the study. To ensure randomization concealment, this independent staff member performed the randomization by opening sealed envelopes, which included the randomized allocation to a gingival displacement material (A: cord or B: paste) and the intervention quadrant (left or right side). The randomization documents had been prepared by the supervising statistician at the Institute of Epidemiology and Medical Biometry, University Ulm.

### 2.7. Blinding

Neither investigator nor proband could be blinded due to the distinguishable gingival displacement materials. A strict blinding protocol was applied for the following evaluation steps.

### 2.8. Statistical Methods

In case of continuous outcomes, both means together with standard deviation and boxplots were used for description. Categorical data were analyzed using absolute frequencies. Next to descriptive analyses, the data were analyzed via two-sample *t* tests for group comparisons (cord vs. paste) in continuous outcomes. In case of unequal variances, the Satterthwaite approximation was used. Group comparisons in categorical outcomes were performed via chi square test. Furthermore, the 95% confidence interval was calculated for primary and secondary outcomes. It was differentiated according to the material (cord/paste) and to the first and second premolar. The results from the statistical tests were considered as significant, if *p* < 0.05. The statistical analysis software was SAS version 9.4 (SAS Institute, Cary, NC, USA) and IBM SPSS Statistics 27 (SPSS Inc., Chicago, IL, USA). All results from the statistical tests have to be interpreted as hypothesis generating only and not as confirmatory, due to the explorative nature of this study. No adjustment for multiple testing was made.

## 3. Results

### 3.1. Participant Flow

**Figure 6:**
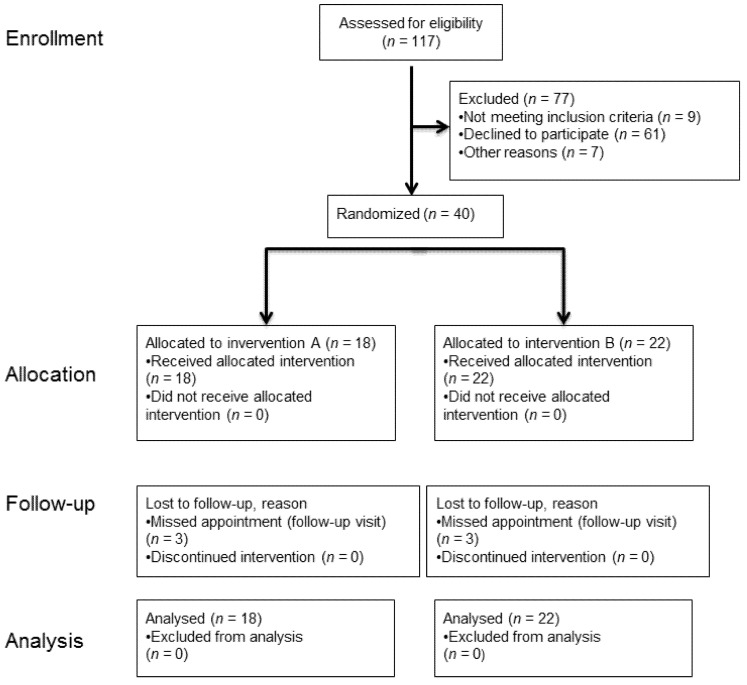
Study flow diagram (according to CONSORT 2010). Intervention A: cord. Intervention B: paste.

To recruit the intended number of subjects for randomization (*n* = 40), 117 individuals were assessed for eligibility (Figure 6). Eighteen subjects were allocated to group A (cord), 22 subjects were allocated to group B (paste). In both groups, three probands missed a follow-up appointment leading to missing values for the secondary outcome “marginal gingiva height”, but not for the other outcomes. Therefore, the data of all 40 probands were available for analyses (no dropout). The intended treatment was carried out in both groups.

### 3.2. Baseline Data

The baseline data were compared between the intervention groups (Table 1). Neither age, nor sex, nor the GI at the intervention visit differed significantly between the groups. For the cord group, the range of age was between 18 and 30 years; for the paste group, the range of age was between 21 and 33 years. Most of the probands were dental students. The baseline data for the absolute sulcus depth and width before gingival displacement are given in the Outcomes Section (Tables 2 and 4). They did not differ significantly between the groups either.

### 3.3. Numbers Analyzed

Data of all 40 probands were included in the analyses, 18 for the cord group and 22 for the paste group. For the primary outcome and the horizontal gingival displacement, all datasets were available. For the marginal gingiva height, six control models were missing due to missed follow-up appointments (A cord: *n* = 3; B paste: *n* = 3).

- One model for each group at the 3-month follow-up visit.
- One model for group A and two models for group B at the 6-month follow-up visit.
- One model for group A at the 9-month follow-up visit.

### 3.4. Outcomes

#### 3.4.1. Vertical Gingival Displacement

The absolute sulcus depth before and after gingival displacement as well as the calculated difference between those two values—representing the vertical gingival displacement—are given in Table 2. Both intervention groups showed comparable baseline data concerning the sulcus depth before gingival displacement. The gingival displacement led to an increase in the absolute sulcus depth by 75% (paste) and 100% (cord), respectively. Comparing the results between both interventions, the cord group showed a somewhat more effective vertical gingival displacement. For the first premolar, this difference reached statistical significance, but not for the second premolar (Table 2). 

##### Comparison to the Prior Study

The comparison of the absolute sulcus depths after gingival displacement between the prior [6] and this study is shown in Table 3. There was a statistically significant difference between the results of both studies, whereas this was not the case for the second premolar of the paste group (*p* = 0.06). For the cord groups applying the same cord system in both studies, the mean absolute sulcus depths were 0.17 mm (first premolar)/0.15 mm (second premolar) (95%-CI (0.04, 0.30) (first premolar)/(0.02, 0.29) (second premolar)) lower in the prior study. For the paste groups applying two different paste systems, the mean absolute sulcus depths were 0.14 mm (first premolar)/0.12 mm (second premolar) (95%-CI (0.04, 0.24) (first premolar)/(0.00, 0.24) (second premolar)) lower for the prior study.

#### 3.4.2. Horizontal Gingival Displacement

The absolute sulcus width before and after gingival displacement as well as the calculated difference between those two values—representing the horizontal gingival displacement—are given in Table 4. Both intervention groups showed comparable baseline data concerning the sulcus width before gingival displacement, as well as comparable data for the sulcus widening (increase by 70%).

**Figure 7:**
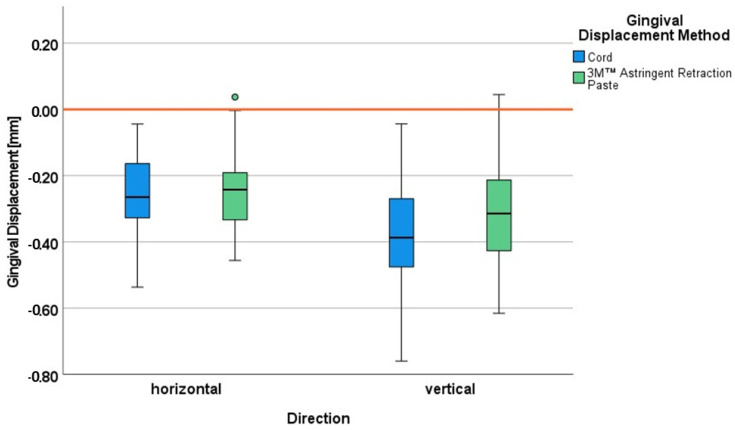
Gingival displacement in horizontal and vertical direction for the cord method and the 3M™ Astringent Retraction Paste; negative value: gain in sulcus width (horizontal)/depth (vertical) after gingival displacement; the circle represents an outlier value (more than 1.5 times the box width away).

Comparing the gingival displacement in both directions (Figure 7), the gingival displacement procedures showed a somewhat stronger effect in the vertical than in the horizontal direction. While the horizontal gingival displacement was not influenced by the gingival displacement method, the cord technique to some extent seemed to be more effective in increasing the sulcular depth.

#### 3.4.3. Marginal Gingiva Height Change

**Figure 8:**
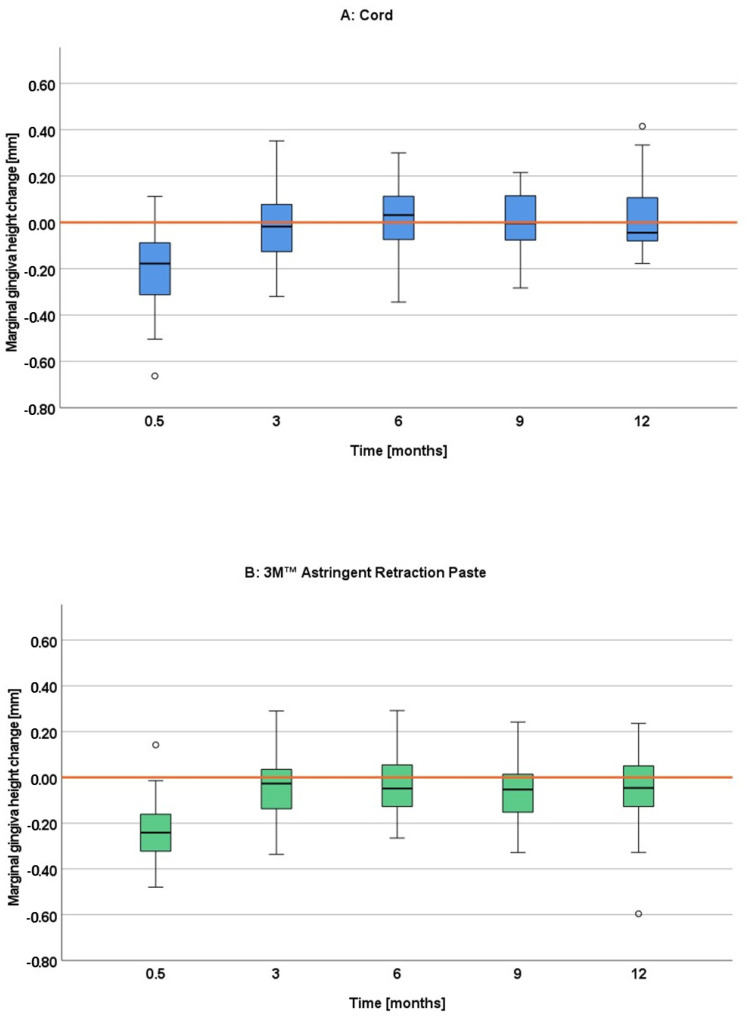
Marginal gingiva height changes between reference and intervention model (0.5 months) and between reference and control models (3, 6, 9, 12 months); negative value: loss of marginal gingiva height compared to the reference model; the circles represent outlier values (more than 1.5 times the box width away).

Both gingival displacement procedures resulted in similar marginal gingiva height changes of about 0.2 mm on average as compared to the reference model at the intervention visit (Figure 8). The marginal gingiva height changes already approached 0 mm after 3 months for both groups. The cord group remained mostly at this level over the follow-up period of 12 months, while the paste group remained slightly below with a mean group difference in the two-sample *t* test of up to 0.12 mm as compared to the cord group. The two-sample *t* test showed significant differences between both groups only at the first premolar after nine and twelve months (Table 5).

### 3.5. Ancillary Analyses

To assess the quality of alignment of the datasets, the RMS (root mean square) error was determined and analyzed. The results proved a high quality (Table 6).

### 3.6. Harms

No severe adverse events (SAE) took place during the study. The palatal marginal gingiva height change analyses revealed values which are considered as clinically unproblematic.

## 4. Discussion

The aim of the study was to quantify the gingival displacement performance of the paste system 3M™ Astringent Retraction Paste in both dimensions (vertical and horizontal) under the condition of mild gingivitis in comparison with the cord technique. Additionally, possible permanent gingival height changes were to be evaluated over 12 months. Under the condition of the mild gingivitis in this study, the horizontal gingival displacement was almost identical for both gingival displacement procedures, whereas the vertical gingival displacement was somewhat more effective for the cord system. The paste system showed slightly more marginal gingiva height loss than the cord system in the last follow-up visits (9 and 12 months) at the first premolar. However, this loss was of such low extent that it cannot be considered as a recession.

Most comparative studies between paste and cord systems only investigated the gingival displacement performance in the horizontal direction, which is also referred to as lateral displacement. Our results are in accordance with those of one of the rare studies investigating the 3M™ Astringent Retraction Paste [10]. They also achieved comparable results for the horizontal gingival displacement by this paste system and a cord technique, although for healthy gingival conditions without any inflammation. In contrast, the several studies comparing other paste systems to the cord technique—again for healthy gingiva—detected worse horizontal gingival displacement for those paste systems, such as Magic Foam Cord or Expasyl [7]. Thus, the 3M™ Astringent Retraction Paste may perform better than other paste systems. The above-mentioned study [10] claims this due to significantly better results in a direct comparison with Expasyl. Besides assumed differences in the exact composition with probably varying consistencies, the application tip differs between both systems. The tip of the 3M™ Astringent Retraction Paste is smaller and designed to correspond to a perio probe [9]. This may support a more effective and complete filling of the sulcus from the bottom and thus potentially more contact area to the tissue to be displaced. The here discussed first clinical evidence of the superiority of the 3M™ Astringent Retraction Paste seems to be in contrast with the conclusions of an in-vitro study, investigating the injection and post injection pressure in an artificial sulcus [16]. As both injection and post injection pressure were significantly lower for the 3M™ Astringent Retraction Paste than for Expasyl, the authors concluded that this system could be less effective in gingival displacement. However, an increased pressure build-up does not necessarily lead to more gingival displacement [29]. There are most likely further decisive factors. A maximum possible contact area of chemically efficient ingredients may lead to short-term drying and contracting of the tissue, and thus overcompensates for the lack of pressure. The first hints from in vivo studies that the 3M™ Astringent Retraction Paste may perform comparable to the cord technique in horizontal direction both for healthy [10] and mildly inflamed gingiva in this study have to be confirmed in further studies.

To assess the magnitude of clinically sufficient horizontal displacement, the minimum value of 0.2 mm became a scientifically recognized threshold [30]. With mean values and respective limits of the 95% confidence interval above this threshold, both cord and paste technique showed a sufficient displacement performance in this study. Thus, the advantages of this paste system such as the high usability in the clinical routine [9,11] and the potentially better hemorrhage control as determined for another aluminum chloride containing paste [13,31], might be exploited more frequently, especially under the condition of mild gingivitis. For the sake of completeness, another potential advantage of aluminum chloride containing pastes shall be mentioned here as derived from the scarce histological literature on this issue. The paste Expasyl caused significantly less cases of disrupted sulcular and junctional epithelium in humans (here: gingiva free of inflammation) than cords impregnated with aluminum chloride, such as used in our study [32]. This advantage could also exist for the aluminum chloride-containing 3M™ Astringent Retraction Paste.

Concerning the gingival displacement in vertical direction, only little evidence is available. The limited scientific data hint to a superiority in the vertical displacement performance of the double cord technique compared with the paste Expasyl for healthy gingival conditions [6,31,33] and mildly inflamed gingiva, although it is much less pronounced because the cord seems to lose most of its advantages under these conditions [6]. For the comparison to the 3M™ Astringent Retraction Paste in this study, the cord also showed a slight superiority in vertical displacement under the condition of mild gingivitis. When biometrically differentiating between both upper premolars, the difference only became significant for the first premolar. We could, with all due caution, attempt to explain this by the slightly different palatal mucosa thickness [34] and a more difficult accessibility, especially for the cord placement, further distally on the second premolar.

The additional analysis of our study comparing the results for the absolute sulcus depth after gingival displacement between this study and the prior study [6] revealed a slight superiority of the 3M™ Astringent Retraction Paste over Expasyl. The same was already deduced with all caution from the scarce literature for the horizontal gingival displacement (see above). The same explanations may be applicable here. However, the comparison also revealed more gain of the absolute sulcus depth by the cord intervention in this study compared to the prior study. As the identical cord intervention and method of quantification were applied for this comparative analysis, the only reasonable explanation for the discrepancy is the influence by the operator. The interventions in this study were performed by another operator than in the prior study, who probably applied the cords with slightly different pressure. The operator also has to be considered as an additional influencing factor for the interpretation of the paste comparison.

Quantifying the gingival displacement in both dimensions with a corresponding methodology in the same study ensures comparability. The new insights thus gained are that the gingival displacement is in a similar range in the horizontal and vertical directions for both cord and 3M™ Astringent Retraction Paste with somewhat more displacement in vertical direction, especially for the cord technique under the condition of mild gingivitis.

The marginal gingiva height change was of such low extent at all four follow-up appointments (3, 6, 9 and 12 months) for both gingival displacement procedures that it cannot be considered as a recession at any time. Looking more in detail, some differences can be found between the techniques and over time. While the marginal gingiva height remained mostly at the baseline level for the cord technique for all four follow up visits, the paste technique remained slightly below indicating a minimal loss. The differences became most pronounced at the 9- and 12-month follow up, reaching significance for the first premolar. This slight tendency of more gingival height loss for a paste system seems to be in contrast to the findings of other studies, where paste systems were more reliable in preserving gingival tissue than cord systems [5,12,13,14,15]. Most studies, however, did not follow-up the gingival height changes over such a long time but only up to three months or less [12,13,14,15]. As the differences were most pronounced at the 9- and 12-month follow-up in our study, the results may also be different in other studies for longer time spans. Furthermore, the results from the other studies, investigating different paste systems, may not be transferable to the 3M™ Astringent Retraction Paste. As already discussed above, there are first hints to a clinical superiority of this paste system in terms of gingival displacement. This might also explain the slight differences regarding the gingival height change here. However, any effect of the two different gingival displacement materials can only be short-termed. One has to keep in mind that due to the clinically irrelevant dimensions of the gingival height loss in this study the findings must not be overestimated.

The etiology of recessions are multifactorial and therefore the exact causes are difficult to capture [35]. The wrong brushing technique as well as orthodontic treatment may be confounding factors, just to mention two of them. Confounding factors, which might be overrepresented in one intervention group, underline the conclusion above, not to overestimate the slight differences between the groups. 

Plaque accumulation, which is especially known to induce inflammation of the gingiva, is an important etiological factor for gingival recessions [35]. Therefore, the question arises of if the artificially induced gingivitis may play a role in the development of marginal gingiva height changes in this study. The extent of inflammation is crucial for the development of those. In an epidemiological study with 710 participants with suboptimal oral hygiene (plaque index according to Silness and Loe between 1 and 2), the group with dental recessions showed severe gingivitis, whereas the group without recessions showed only moderate gingivitis [35]. Against this background and since we only produced mild gingivitis by a comparatively short induction period and we performed regular professional tooth cleaning directly afterward and during the further course of the study, it is rather unlikely that the induced gingivitis led to marginal gingiva height changes. Another reason that speaks against the temporary gingivitis causing marginal gingiva height changes, arises from the specific dental sites investigated in the study. In an epidemiological study with 575 participants deprived of prophylactic dental care, it was shown that the upper premolars’ palatal sites were highly resistant to recession development. While participants from the corresponding age group to our study showed recessions at other dental sites, they were almost free of any recession at these sites [36]. The gingiva at these sites could, of course, also show a comparably high resistance against the possible development of marginal gingiva height changes by the gingival displacement measures.

The design of this study was, in few basic features, similar to that of a prior study performed at the same study center, whose design has already been thoroughly discussed [6]. The design was adapted to the current scientific question as formulated at the end of the introduction section. The analyses were further developed and extended, especially by the analysis for the gingival displacement in the horizontal direction. Most studies cut the plaster models and performed measurements in the section plane either directly with a microscope and metric scale [7,13,14] or with a software after taking digital microscope images [7,8,10,19]. Some other studies performed the measurements at predefined sites at the impression itself [7,24,31]. With those techniques, the sulcus width is evaluated at few sulcular sites—often only one site. With the newly developed computer-assisted method of this study, the sulcus width is evaluated along the complete palatal half of the tooth, making it more robust to variations across the sulcus. 

The computer analysis method allows for a variety of further possibilities for quantifying gingival displacement up to the determination of the 3D volume of the displacement. For the following reasons, however, we have decided to maintain the classical linear determination. The displacement in vertical direction is essential to capture subgingival preparation margins. The displacement in horizontal direction is essential to achieve sufficient thickness of the impression material to avoid distortion or tearing off [1,30]. The measurement of the enhancement of the area [4,20] or the 3D volume does not give differentiated information about the displacement in those two crucial directions.

When comparing the values between studies, caution must be exercised, and not only due to the above-mentioned varying methodologies, which may lead to different values. Additionally, the definition of the outcome varies between the studies. In some studies, only the absolute sulcus width after gingival displacement is presented [13,14]. The authors of other studies calculated the difference and thus presented the horizontal gingival displacement achieved by the retraction technique [8,19]. In our study, we presented both absolute values as well as the difference between them, thus the actual gingival displacement. 

For the analyses of the gingival displacement in vertical direction, we applied the same procedure as described above, i.e., we presented both absolute sulcus depths (before/after gingival displacement) and the actual gingival displacement. The vertical gingival displacement has rarely been investigated. Again, caution is advised when comparing the results between different studies due to even more distinct differences in the definition of the outcome. The vertical gingival displacement corresponds to the apical displacement of the gingival crest (not the sulcus) in some studies [24,37,38]. We also measured this displacement in our study in the course of the marginal gingiva height change analyses. These are the values at the intervention visit (0.5 month). Those differed from the values of the vertical gingival displacement, especially for the cord technique. The differences clearly show that a concordant definition of outcomes is a prerequisite for comparability.

We performed the study on unprepared teeth. The marginal contour differs between prepared and unprepared teeth, depending on the extent of preparation. An influence of this contour on the effect of gingival displacement techniques on the gingiva is unlikely, especially with the recommended preparation techniques. If subgingival preparation is necessary, e.g., for esthetic reasons, the position of the finish line should be only minimally subgingival without jeopardizing the supracrestal attachment (former: biological width), because this would cause histological changes with an apical shift of the periodontal structures [39]. Multiple clinical studies on gingival displacement performance accordingly performed preparation with finish lines at the height of the free gingival margin [13,14,19,40]. With this location of the finish line, the contour above the finish line is less relevant, as the main area of action of the gingival displacement techniques, where the gingival expansion takes place in vertical and horizontal direction is alongside and apical to the finish line. This area is identical for both prepared and unprepared teeth. Therefore, the conduct of clinical studies on gingival displacement performance has been established for many years on unprepared teeth [3,7,18,20] in addition to prepared teeth. Thus, the advantages of studies on unprepared teeth can be exploited, such as a more feasible recruitment, if specific teeth (e.g., upper premolars) shall be investigated. Another advantage of using unprepared teeth is for studies that investigate the effect of gingival displacement techniques on marginal gingiva height changes, such as is investigated in this study. Thus, the potential confounding factor of inserting a fixed dental prosthesis—especially in case of suboptimal prosthesis design—on this outcome is excluded.

We performed the study under the condition of mild gingivitis, which was induced. For the induction of artificial gingivitis, the following very well researched procedure was used. A pre-induction phase with professional tooth cleaning was followed by an induction phase with abandonment of oral hygiene. The final resolution phase terminated the gingivitis by resumption of oral hygiene. According to a meta-analysis of clinical trials, typical induction phases for artificial gingivitis range from 10 days (4 of 22 included studies) to 28 days. Twenty-one days were the most common period [41]. Tracking inflammation using a defined clinical inflammation index (mostly gingival index by Loe [28]) shows a continuous increase in index values up to the maximum period of 21 days studied here [42,43,44], with up to severe inflammation obtained at 21 days. We deliberately chose a shorter induction period of 10 to 14 days because we were aiming for only mild gingivitis, where an impression is still reasonable. Thus, the definition of mild gingivitis in our study results from the choice of the induction period of only 10 to 14 days according to the well-established methodology for establishing artificial gingivitis.

We did not gain tissue samples from the probands to perform histological preparations to histologically confirm the inflammation. In this study design, it was not justifiable for ethical reasons. Obtaining gingival biopsies from healthy teeth solely for the purpose of research can only be justified if it is absolutely necessary and the inflammatory state cannot be ensured by other means. We did not consider this to be the case here. There is an extremely well-researched and functioning tool in the artificial gingivitis methodology for the safe establishment of gingivitis in different degrees of severity from mild to severe, depending on the induction phase, as described above.

We can draw the following conclusion for the clinical application from this study: due to the similar performance of both gingival displacement techniques under the condition of mildly inflamed gingiva, the advantages of the 3M™ Astringent Retraction Paste—its high usability and potentially better hemorrhage control as derived from the literature—might be exploited more frequently in such cases.

One of the study’s limitations is the range of age of the actually included patients. They belonged to a specific age group with a mean of 24 years for both interventions (see baseline data). Therefore, our data applied to this age group and might be transferable to others only with caution, if differences exist. To detect possible differences, a further study including stratification by age group would be necessary. This can either be performed by high sample sizes or by risking an underpowered study. Another limitation results from the specific dental sites investigated in the study. As explained above, the literature shows a high resistance of the oral sites of the upper premolars against the development of recessions in general. Thus, the gingiva at these sites could also show a comparably high resistance against the possible development of marginal gingiva height changes by gingival displacement measures. To evaluate this issue, a further study performing the same procedures at different dental sites would be necessary.

## 5. Conclusions

Under the condition of mildly inflamed gingiva, the following conclusions can be drawn for the gingival displacement by the 3M™ Astringent Retraction Paste and the double cord technique with aluminum chloride prior to conventional impression making:

- The performance was comparable for both techniques in the horizontal direction.
- The performance was only somewhat better for the cord technique in the vertical direction.
- The magnitude of displacement was in a similar range in both directions, with somewhat higher values in the vertical direction.
- Marginal gingiva height changes were of such low extent during the follow-up period of 12 months with only minimally higher values for the paste that they cannot be considered as clinically relevant recessions.
- An additional analysis including data from a prior study [6] revealed first clinical hints of a superiority of the vertical gingival displacement of the 3M™ Astringent Retraction Paste when compared to Expasyl.

## Figures and Tables

**Table 1:** Baseline data for group A (cord) and group B (paste).

	Group A (Cord) (Mean ± SD)	Group B (Paste) (Mean ± SD)	*p* Value
Age	24.0 ± 2.5	24.2 ± 2.9	0.89 (n.s.) ^1^
Sex (m:f)	10:8	10:12	0.53 (n.s.) ^2^
GI (Intervention, Visit 4)	1.62 ± 0.65	1.79 ± 0.42	0.36 (n.s.) ^1^

^1^ Two-sample *t* test for independent samples; ^2^ Chi square test; GI: Gingival index; SD: standard deviation; n.s.: not significant (*p* ≥ 0.05).

**Table 2:** Absolute sulcus depth before (ASDB) and after (ASDA) gingival displacement and vertical gingival displacement for the cord and the paste technique.

	Absolute Sulcus Depth before Gingival Displacement (ASDB) (mm) Mean ± SD (LL/UL)	Absolute Sulcus Depth after Gingival Displacement (ASDA) (mm) Mean ± SD (LL/UL)	Vertical Gingival Displacement (Difference ASDB—ASDA) Mean ± SD (LL/UL)
Cord (*n* = 36; both premolars)	0.41 ± 0.09 (0.38/0.44)	0.79 ± 0.19 (0.73/0.86)	−0.39 ± 0.19 (−0.45/−0.32)
Paste (*n* = 44; both premolars)	0.39 ± 0.11 (0.36/0.42)	0.70 ± 0.17 (0.65/0.75)	−0.31 ± 0.15 (−0.36/−0.27)
Cord versus paste: *p* value ^1^ (1st premolar/2nd premolar)	0.56 (n.s.)/0.67 (n.s.)	0.02 (s.)/0.39 (n.s.)	0.04 (s.)/0.47 (n.s.)

^1^ Two-sample *t* test for independent samples; SD: standard deviation; LL: lower limit of 95% confidence interval; UL: upper limit of 95% confidence interval; s.: significant (*p* ˂ 0.05); n.s.: not significant (*p* ≥ 0.05).

**Table 3:** Comparison of the absolute sulcus depth after gingival displacement between the prior study [6] and this study.

	Absolute Sulcus Depth after Gingival Displacement (mm) Prior Study (WGMI) Cord; *n* = 17 Expasyl Paste; *n* = 17 (Mean ± SD)	Absolute Sulcus Depth after Gingival Displacement (mm) Cord; *n* = 18 3M™ Astringent Retraction Paste; *n* = 22 (Mean ± SD)	*p* Value ^1^
Cord; 1st premolar	0.65 ± 0.18	0.81 ± 0.20	0.01 (s.)
Cord; 2nd premolar	0.62 ± 0.20	0.77 ± 0.18	0.02 (s.)
Paste; 1st premolar	0.54 ± 0.16	0.68 ± 0.14	0.01 (s.)
Paste; 2nd premolar	0.60 ± 0.15	0.72 ± 0.20	0.06 (n.s.)

^1^ Two-sample *t* test for independent samples; SD: standard deviation; s.: significant (*p* ˂ 0.05); n.s.: not significant (*p* ≥ 0.05).

**Table 4:** Absolute sulcus width before (ASWB) and after (ASWA) gingival displacement and horizontal gingival displacement for the cord and the paste technique.

	Absolute Sulcus Width before Gingival Displacement (ASWB) (mm) Mean ± SD (LL/UL)	Absolute Sulcus Width after Gingival Displacement (ASWA) (mm) Mean ± SD (LL/UL)	Horizontal Gingival Displacement (Difference ASWB—ASWA) Mean ± SD (LL/UL)
Cord (*n* = 36; both premolars)	0.36 ± 0.11 (0.32/0.39)	0.61 ± 0.13 (0.57/0.66)	−0.26 ± 0.12 (−0.30/−0.22)
Paste (*n* = 44; both premolars)	0.36 ± 0.10 (0.33/0.39)	0.61 ± 0.15 (0.56/0.65)	−0.25 ± 0.11 (−0.28/−0.21)
Cord versus paste: *p* value ^1^ (1st premolar/2nd premolar)	0.88 (n.s.)/0.86 (n.s.)	0.65 (n.s.)/0.82 (n.s.)	0.69 (n.s.)/0.92 (n.s.)

^1^ Two-sample *t* test for independent samples; SD: standard deviation; LL: lower limit of 95% confidence interval; UL: upper limit of 95% confidence interval; n.s.: not significant (*p* ≥ 0.05).

**Table 5:** Comparison of marginal gingiva height change between cord and paste group.

Time Interval to Reference Impression (Months)	*p* Value ^1^ 1st Premolar	*p* Value ^1^ 2nd Premolar
0.5 (Intervention Visit)	0.73 (n.s.)	0.35 (n.s.)
3	0.42 (n.s.)	0.84 (n.s.)
6	0.08 (n.s.)	0.46 (n.s.)
9	0.01 (s)	1.00 (n.s.)
12	0.04 (s)	0.68 (n.s.)

^1^ Two-sample *t* test for independent samples. s.: significant (*p* ˂ 0.05); n.s.: not significant (*p* ≥ 0.05).

**Table 6:** Root mean square (RMS) error for the vertical gingival displacement analyses, horizontal gingival displacement analyses and for the marginal gingiva height change analyses (alignment of all first control models to the reference models).

	Vertical Gingival Displacement	Horizontal Gingival Displacement	Marginal Gingiva Height Change
Mean RMS Error ± SD (µm)	9.3 ± 4.4	11.7 ± 8.4	9.1 ± 3.4
Percentage of RMS Errors Below the Threshold of 32 µm	100%	95%	100%

SD: standard deviation.

## Data Availability

The data presented in this study are available on request from the corresponding author.
